# Transparent and Robust *All*-Cellulose Nanocomposite Packaging Materials Prepared in a Mixture of Trifluoroacetic Acid and Trifluoroacetic Anhydride

**DOI:** 10.3390/nano9030368

**Published:** 2019-03-05

**Authors:** Susana Guzman-Puyol, Luca Ceseracciu, Giacomo Tedeschi, Sergio Marras, Alice Scarpellini, José J. Benítez, Athanassia Athanassiou, José A. Heredia-Guerrero

**Affiliations:** 1 Smart Materials, Nanophysics, Istituto Italiano di Tecnologia, Via Morego, 30, 16163 Genova, Italy; giacomo.tedeschi@iit.it; 2 Materials Characterization Facility, Istituto Italiano di Tecnologia, Via Morego, 30, 16163 Genova, Italy; luca.ceseracciu@iit.it (L.C.); sergio.marras@iit.it (S.M.); 3 Electron Microscopy Facility, Istituto Italiano di Tecnologia, Via Morego, 30, 16163 Genova, Italy; alice.scarpellini@iit.it; 4 Instituto de Ciencia de Materiales de Sevilla, Centro mixto CSIC-Universidad de Sevilla, Calle Americo Vespucio 49, Isla de la Cartuja, 41092 Sevilla, Spain; benitez@icmse.csic.es

**Keywords:** *All*-cellulose nanocomposites, trifluoroacetic acid, trifluoroacetic anhydride, transparency, robustness, packaging material

## Abstract

*All*-cellulose composites with a potential application as food packaging films were prepared by dissolving microcrystalline cellulose in a mixture of trifluoroacetic acid and trifluoroacetic anhydride, adding cellulose nanofibers, and evaporating the solvents. First, the effect of the solvents on the morphology, structure, and thermal properties of the nanofibers was evaluated by atomic force microscopy (AFM), X-ray diffraction (XRD), and thermogravimetric analysis (TGA), respectively. An important reduction in the crystallinity was observed. Then, the optical, morphological, mechanical, and water barrier properties of the nanocomposites were determined. In general, the final properties of the composites depended on the nanocellulose content. Thus, although the transparency decreased with the amount of cellulose nanofibers due to increased light scattering, normalized transmittance values were higher than 80% in all the cases. On the other hand, the best mechanical properties were achieved for concentrations of nanofibers between 5 and 9 wt.%. At higher concentrations, the cellulose nanofibers aggregated and/or folded, decreasing the mechanical parameters as confirmed analytically by modeling of the composite Young’s modulus. Finally, regarding the water barrier properties, water uptake was not affected by the presence of cellulose nanofibers while water permeability was reduced because of the higher tortuosity induced by the nanocelluloses. In view of such properties, these materials are suggested as food packaging films.

## 1. Introduction

The massive use of petroleum-based plastics in disposable food packaging materials has triggered a global social concern, mainly because of the pollution derived from their synthesis and the related littering problems [1,2,3,4]. For this reason, a new model of economic activity, namely “circular economy,” has emerged [5,6,7]. Among the different proposals of a circular economy applied to these plastics, the production of bio-based plastics from alternative feedstocks such as agro-food by-products and naturally occurring biopolymers is a strategy that is attracting the attention of researchers [8,9,10,11,12,13,14,15]. Cellulose is the most abundant polymer on Earth with an annual biomass production of about 1.5 × 10^12^ tons per year, being, hence, one of the most promising bio-renewable resources for reducing and replacing the huge amount of petroleum-based plastics. Cellulose shows full biodegradability in soil and seawater over short times, is lightweight and has excellent mechanical strength [16,17,18]. Among the vast group of cellulose materials, *all*-cellulose composites (ACCs) are a category of particular interest. These composites are materials in which both the reinforcement and the matrix are cellulose [19,20]. The use of the same material acting as matrix and reinforcement increases the compatibility between the phases and, therefore, the mechanical properties of the composite [21]. Two different procedures have been reported for the preparation of *all*-cellulose composites [20,22]. The first is a two-step method in which cellulose is partially dissolved and then regenerated in the presence of undissolved cellulose. Thus, regenerated and undissolved cellulose fractions may come from different natural origins [23]. The second procedure consists of a one-step method where the surface of cellulose is partially dissolved and regenerated in situ to create a matrix around the non-dissolved portion. The most common solvents employed in these processes are LiCl/DMAc, *N*-Methylmorpholine *N*-oxide (NMMO), NaOH, and the ionic liquid 1-butyl−3-methylimidazolium chloride [20]. Nevertheless, partial and slow cellulose dissolution and non-recyclability have limited the use of these solvents on an industrial scale. In any case, the dissolution process described above for both methods is followed by subsequent solvent removal, cellulose regeneration, and drying [20]. The result of such a process is an *all*-cellulose composite with exceptional mechanical properties [24], optical transparency [25], and improved barrier properties [22] with respect to regenerated cellulose, as well as full biodegradability [26]. *All*-cellulose composites are used in a wide range of applications such as the reinforcement of other polymers, substitution of bone and cartilage materials, the fabrication of electro-active paper, sensors and electrical displays, and the production of biodegradable food packaging materials and mulching films for agriculture [26,27,28,29,30,31,32,33,34,35,36,37].

Trifluoroacetic acid (TFA) is one of the non-aqueous derivatizing solvents for cellulose [38,39,40,41,42,43,44]. The dissolution of cellulose by TFA might not occur in the absence of a chemical reaction [38]: cellulose is trifluoroacetylated selectively in the C6-hydroxyl groups [39]. This derivative is readily hydrolyzed in water, water vapor, or the moisture in the air, forming amorphous and transparent cellulose films [11,38,40]. Trifluoroacetic acid is a naturally occurring organic acid and biodegradable by microbial action [38,45,46]. Moreover, it is recyclable by distillation due to its high volatility and is miscible with many organic solvents and water [11]. TFA has been recently used to fabricate bioplastics from microcrystalline cellulose and plant wastes, as well as blends of cellulose with seaweeds, silk, nylon, poly(methyl methacrylate), and poly(vinyl alcohol) [11,47,48,49,50]. When TFA is combined with trifluoroacetic acid anhydride (TFAA), a reactive mixture that allows the acylation of cellulose and cellulose derivatives with carboxylic acids is generated [51,52].

In this work, we prepared *all*-cellulose nanocomposites with potential application in food packaging by using a simple method consisting of dissolving cellulose in a trifluoroacetic acid:trifluoroacetic anhydride (2:1, v:v) mixture and subsequent addition of different cellulose nanofibers dispersed in chloroform. The effect of the solvent in the nanocelluloses was investigated. In addition, the influence of different percentages of these nanofillers on the morphology, optical, mechanical, thermal, and hydrodynamic properties of the nanocomposites was assessed. Furthermore, a model was developed to analyze the mechanical properties.

## 2. Materials and Methods

### 2.1. Materials

Trifluoroacetic acid (TFA), trifluoroacetic anhydride (TFAA), microcrystalline cellulose (MCC) from cotton linters (crystallinity ~79%), and chloroform were purchased from Sigma-Aldrich (St. Gallen, Switzerland) and used without additional purification. Cellulose nanofibers (two types: one nominally shorter than the other and labeled as sNF and lNF, respectively) were purchased from Nanografi (Ankara, Turkey) and used as received. These nanofibers were prepared from wood pulp by using mechanical methods and commercialized as dry powders.

### 2.2. Fabrication of All-Cellulose Nanocomposites

The preparation of *all*-cellulose nanocomposites was carried out as follows: first, MCC (450 mg) was dissolved in 30 mL of TFA:TFAA (2:1, v:v) in a 50 mL closed flask and stirred at 50 °C until the solution was completely clear (~1 h). Later, cellulose nanofibers (4.5, 22.5, 45, 90 and 135 mg) were mixed with 30 mL chloroform and dispersed by three consecutive 30 s ultrasound cycles using a 3.2 mm diameter tapered microtip at 10% amplitude attached to a VCX 750 ultrasonic processor (Sonics & Materials, Inc., Newtown, CT, USA). Then, both solutions were blended together and cast in glass Petri dishes. The mixture of solvents was completely evaporated after 1 day under an aspirated hood, originating freestanding films. Pure cellulose films were also prepared as a control using the same protocol in order to study the role of the nanofibers as reinforcement of the cellulose matrix. Similarly, to analyze the effect of the solvents on the nanocelluloses, the nanofibers were subjected to the above treatment but without adding MCC. All samples were stored at 44% relative humidity (RH) for 7 days before analysis to ensure the reproducibility of the measurements. Table 1 summarizes the label and the final composition of the different samples.

### 2.3. Morphological Characterization

Atomic force microscopy (AFM) images were acquired using a Nanotec microscope (Nanotec, Madrid, Spain) in low amplitude dynamic mode. Levers used were Nanosensors PPP-NCH (NanoWorld AG, Neuchâtel, Switzerland) with a tip radius curvature less than 10 nm and a resonance frequency of 295 kHz (29 N/m force constant). Samples were prepared from sonicated diluted dispersions (0.1 mg/30 mL water) by placing a 10 µL drop on a freshly cleaved mica muscovite piece (~1 cm^2^) and allowing to dry overnight inside a Petri dish. The width and length of the nanofibers were measured with WSxM software [53]. Approximately 100 measurements were taken to obtain each width and length distribution.

High-resolution scanning electron microscopy (SEM) imaging was carried out using a JEOL JSM 7500FA (Jeol, Tokyo, Japan) equipped with a cold field-emission gun (FEG), operating at 15 kV acceleration voltage. The samples were coated with a 10 nm thick film of carbon using an Emitech K950X high-vacuum turbo system (Quorum Technologies Ltd., East Sussex, Lewes, UK). Imaging was performed with the secondary electrons to analyze the morphology of the samples. 

### 2.4. Optical Characterization 

Transparency was determined as the normalized transmittance according to the standard ASTM D1746 by using a ultraviolet (UV) spectrophotometer Varian Cary 6000i (USA) [54]. For this, samples were cut into rectangular pieces and placed directly in the spectrophotometer test cell. An empty test cell was used as a reference. Five measurements were taken from different samples and the results were averaged to obtain a mean value. Normalized transmittance, in percentage, was calculated as indicated below:

$$Normalized\;transmittance\;\left(\%\right)=\frac{\log\%T}{b}\times 100$$

where %*T* is the transmittance at 600 nm and *b* is the thickness of the sample (mm).

### 2.5. Structural Characterization

X-ray diffraction (XRD) patterns were recorded on a Rigaku SmartLab X-ray powder diffractometer equipped with a 9 kW CuKα rotating anode (Rigaku, Tokyo, Japan), operating at 40 kV and 150 mA. A Göbel mirror was used to convert the divergent X-ray beam into a parallel beam and to suppress the Cu Kβ radiation. The specimens were analyzed at room temperature using a zero diffraction quartz sample holder. XRD data analysis was carried out using PDXL 2.1 software from Rigaku. The crystallinity index (*CrI*) was determined by using the empirical method proposed by Segal et al. [55]:

$$CrI\left(\%\right)=\frac{I-I^{\prime }}{I}\times 100$$

where *I* is the intensity of the peak assigned to (002) crystal plane of cellulose located at 21−23° and *I*′ is the intensity of the diffractogram of the amorphous cellulose at 18−19°. In addition, the crystallite size of cellulose (*D*) was estimated by Scherrer’s Equation:

$$D=\frac{K\;\lambda }{\beta \cos\theta }$$

where K is a constant of value 0.94, *λ* is the X-ray wavelength (0.15418 nm), *θ* is the diffraction angle for the (200) plane, and *β* is the peak width at half the maximum intensity (calculated from peak deconvolution when necessary).

### 2.6. Mechanical Characterization

The mechanical properties of the films were measured by uniaxial tensile tests on a dual column Instron 3365 universal testing machine. Dog-bone-shaped samples were stretched at a rate of 5 mm/min. All the stress–strain curves were recorded at 25 °C and 44% RH. Ten measurements were conducted for each sample and the results were averaged to obtain a mean value. From the stress–strain curves, Young’s modulus, yield stress, elongation at the break, and fracture energy (area below the curve) were calculated.

### 2.7. Thermal Characterization

The thermal degradation behavior of the nanocelluloses was investigated by a standard thermogravimetric analysis (TGA) method using a TGA Q500 from TA Instruments (New Castle, DE, USA). Measurements were performed using 3–5 mg of sample in an aluminum pan under inert N_2_ atmosphere with a flow rate of 50 mL/min in a temperature range from 30 to 600 °C with a heating rate of 5 °C/min. The weight loss and its first derivative were recorded simultaneously as a function of time/temperature.

### 2.8. Water Uptake and Permeability

For water uptake measurements, samples were first dried by conditioning in a desiccator until no change in sample weight was measured. Dry samples were weighed (~30 mg) on a sensitive electronic balance and, then, placed in a 100% relative humidity (RH) chamber at 25 °C. Once the equilibrium was reached, each sample was again weighed and the amount of adsorbed water was calculated as the difference with the initial dry weight. Three measurements were taken and the results were averaged to obtain a mean value. Water uptake, in percentage, was calculated as indicated below:

$$Water\;uptake\;\left(\%\right)=\frac{m_{f}-m_{0}}{m_{0}}\times 100$$

where *m_f_* is the sample weight at 100% RH and *m*_0_ is the sample weight at 0% RH.

Water vapor permeability (WVP) of *all*-cellulose nanocomposites was determined at 25 °C and under 100% relative humidity gradient (ΔRH %) according to the ASTM E96 standard method [56,57]. Then, 400 µL of deionized water (which generates 100% RH inside the permeation cell) was placed in each test permeation cell (7 mm inside diameter, 10 mm inner depth). *All*-cellulose composites were cut into circles and mounted on the top of the permeation cells. The permeation cells were placed in a 0% RH desiccator with anhydrous silica gel used as a desiccant agent. The water transferred through the film was determined from the weight change of the permeation cell every hour over 7 h using an electronic balance (0.0001 g accuracy). The weight loss of the permeation cells was plotted as a function of time. The slope of each line was calculated by linear regression and the water vapor transmission rate (WVTR) was determined as below:

$$WVTR\;\left(g/\left(m^{2}\cdot day\right)\right)=\frac{Slope}{Area\;of\;the\;film}$$

WVP measurements were replicated three times for each sample. The WVP value was calculated as follows:

$$WVP\;\left(g/\left(m\cdot day\cdot Pa\right)\right)=\frac{WVTR\times l\times 100}{p_{s}\times \Delta RH}$$

where *l* (m) is the film thickness measured with a micrometer with 0.001 mm accuracy, *ΔRH* (%) is the percentage relative humidity gradient, and *p_s_* (Pa) is the saturation water vapor pressure at 25 °C (3168 Pa).

## 3. Results and Discussion

### 3.1. Effect of TFA/TFAA Mixture on the Cellulose Nanofibers

**Figure 1:**
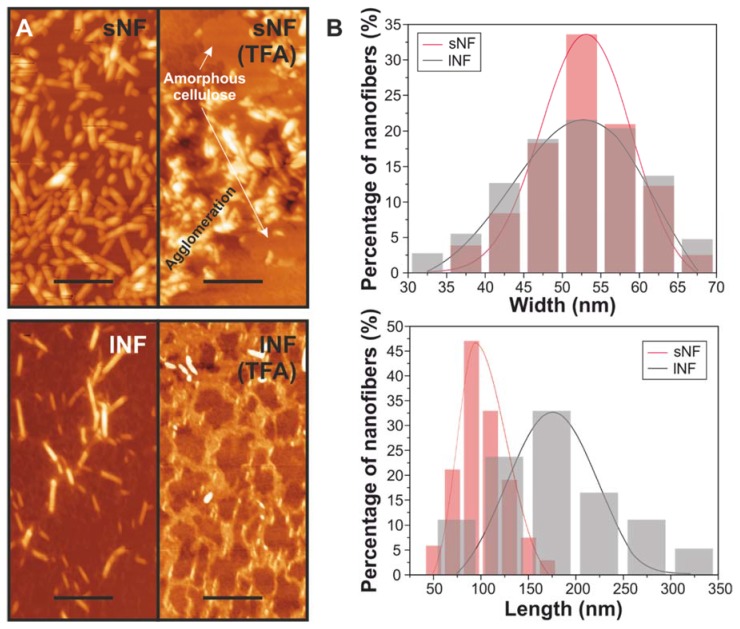
(**A**) Atomic force microscopy (AFM) topographies of short nanofibers (sNF) and long nanofibers (lNF) before and after the solvent treatment. The amorphous domains and agglomerations in the topography of the treated lNF are indicated. Scale bar = 400 nm. (**B**) Histograms showing the width (**top**) and length (**bottom**) distributions of the cellulose nanofibers: sNF (red), lNF (black).

Short and long nanofibers (sNF and lNF, respectively) before and after TFA:TFAA treatment were morphologically characterized by AFM (Figure 1). Figure 1A shows the AFM topographies of the pristine and treated cellulose nanofibers. Both types of pristine nanocelluloses exhibited a fiber morphology. The distribution of widths and lengths for each kind of nanofiber is displayed in Figure 1B. While the widths of both nanocelluloses were very similar with a maximum at ~53 nm (although sNF showed a narrower distribution), the values of the length were different: the maximums were ~100 and ~175 nm for sNF and lNF, respectively. Over again, the distribution of sNF was narrower than the lNF. Interestingly, the mixture of solvents produced important changes in the morphology of the cellulose nanofibers (Figure 1A). Broadly, agglomerations of the nanoparticles and flat islands of height ~2 nm were observed. Such islands could be produced by a partial solution of the cellulose by the TFA/TFAA mixture and the formation of flat, amorphous cellulose when solvents were evaporated. Similar flat and featureless AFM topography images with roughness <2 nm were obtained for cellulose bioplastics prepared in TFA [11].

**Figure 2:**
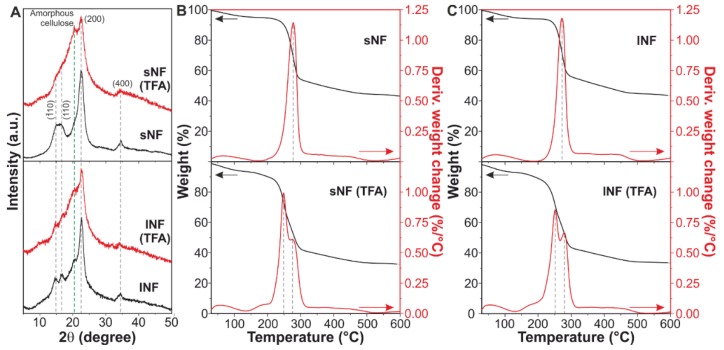
(**A**) X-ray diffraction (XRD) patterns of sNF and lNF before and after the trifluoroacetic acid/trifluoroacetic acid anhydride (TFA/TFAA) treatment. Main assignments are included. (**B**,**C**) Thermogravimetric analysis (TGA) curves and their corresponding derivatives of sNF and lNF, respectively, before and after the TFA/TFAA treatment.

The crystallinity of the cellulose nanofibers was evaluated by XRD (Figure 2A). The pattern of the pristine nanofibers was typical of cellulose I structure [58]. Main peaks were assigned to the following crystalline planes: ($1\bar{1}$0) at ~15°, (110) at ~17°, (200) at ~23°, and (400) at ~35°, while a minor amorphous contribution was observed at ~21° [59]. After the solvent treatment, crystalline peaks were partially masked by the amorphous one. In fact, the CrI decreased from ~58 and ~45% for pristine sNF and lNF, respectively, to ~13 and ~24% for sNF and lNF after the TFA/TFAA treatment. Moreover, the crystallite size of cellulose was reduced from ~4.0 and ~4.3 nm for pristine sNF and lNF, respectively, to ~2.9 and ~3.1 nm for sNF and lNF after the TFA/TFAA treatment. Hence, the mixture of TFA and TFAA can partially dissolve the cellulose nanofibers, decreasing the crystallinity and the crystallite size of cellulose and originating amorphous cellulose, as observed in AFM images.

The effect of the solvent treatment in the thermal properties of the nanofibers was analyzed by TGA (Figure 2B,C). Pristine sNF and lNF showed a similar behavior with a single weight loss of ~56% at ~275 °C. On the other hand, after the solution in TFA/TFAA, both types of nanocelluloses showed two thermal events: a weight loss of ~30% at ~250 °C and another of ~17% at ~275 °C. The thermal degradation at a lower temperature can be related to the partial hydrolysis of amorphous and lower molecular weight cellulose domains that appear after the solvent treatment [60,61], while the second one can be ascribed to the part of the nanocelluloses unaffected by the acid and the anhydride.

### 3.2. Optical and Morphological Characterization of the Nanocomposites

**Figure 3:**
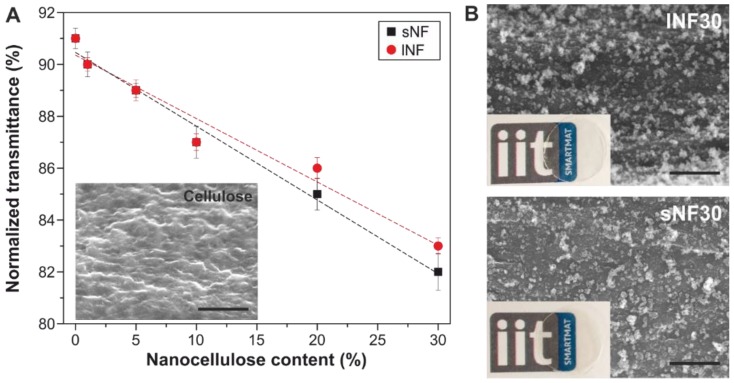
(**A**) Normalized transmittance as a function of nanocellulose content. Inset: HR-SEM cross-section image of a cellulose sample. (**B**) HR-SEM cross-section images of lNF30 and sNF30 samples. Scale bar: 500 nm.

Transparency is an important feature of food packaging materials since it allows the consumers a visual and direct inspection of the food and it is usually characterized by UV–Vis spectroscopy [10,62]. Figure 3A shows the transparency (i.e., the normalized transmittance calculated from these spectra as the ratio of the corresponding transmittance at 600 nm and the film thickness) for all the samples as a function of the nanocellulose content. In general, the transparency values were higher than 80%, which is considered as the lower limit for good transparency [54]. As observed, there was a relationship between the normalized transmittance and the nanocellulose content independent of the type of cellulose nanofiber used. Values ranged from ~91% for cellulose to ~84 and ~83% for sNF30 and lNF30, respectively. Most likely, this decrease can be related to a higher light scattering induced by the cellulose nanoparticles. To corroborate this, the distribution of the nanocellulose fillers in the cellulose matrix was characterized by HR-SEM (Figure 3B). The cross-sections of lNF30 and sNF30 are shown in Figure 3B. While cellulose displayed a smooth, homogeneous topography (inset Figure 1A), lNF30 and sNF30 exhibited rougher cross-sections with motifs of few tens of nanometers that can be attributed to folded or aggregated nanocelluloses.

### 3.3. Mechanical Characterization of the Nanocomposites

**Figure 4:**
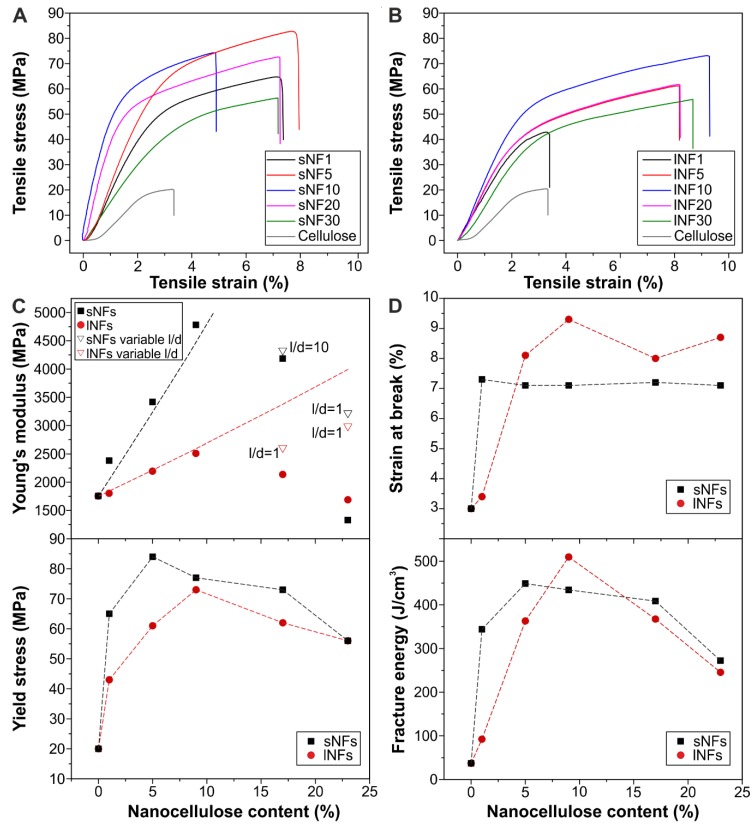
(**A**,**B**) Stress–strain curves of *all*-cellulose nanocomposites prepared with short and long nanofibers, respectively. (**C**) Experimental Young’s modulus as a function of nanocellulose content; the dashed line indicates the analytical model for lNF composites. As the nanocellulose content is increased, the model does not fit the experimental data, even if aggregation is accounted for as a variation of the aspect ratio (hollow points). (**D**–**F**) Yield stress, strain at the break, and fracture energy values as a function of nanocellulose content.

Stress–strain curves of *all*-cellulose nanocomposites are shown in Figure 4A,B. In general, the curves were typical of rigid materials with high stresses at the break and low values of elongation at the break. A strong reinforcement effect due to the addition of nanocelluloses was clearly observed. The shape of the curves depended on the amount of nanocellulose and was unrelated to the type of nanofiller used. Figure 4C shows Young’s modulus values of the *all*-cellulose films. Initially, Young’s modulus increment followed a linear trend from cellulose (~1750 MPa) to a 10 wt.% nanocellulose concentration (~4783 MPa for sNF10 and ~2510 MPa for lNF10) but decreased progressively from that content with either cellulose nanofibers. The composites produced with sNF nanofibers were much stiffer than the longer ones. This is counterintuitive as longer particles are expected to better transfer load from the matrix and to form a more interconnected network. The lower rigidity can be attributed to a higher tendency to aggregation or to lower initial modulus of the longer nanofibers compared to the shorter ones. Both aspects were evaluated by modeling the composite modulus of the lNFs materials through the classic Mallick’s model for laminae with randomly dispersed fibers [63]:

(1a) $$E_{c}=\left[\frac{3}{8}\frac{1+2\left(l/d\right)\eta_{L}V_{f}}{1-\eta_{L}V_{f}}+\frac{5}{8}\frac{1+2\eta_{T}V_{f}}{1-\eta_{T}V_{f}}\right]E_{m}$$

(1b) $$\eta_{L}=\frac{E_{f}/E_{m}-1}{E_{f}/E_{m}+2\left(l/d\right)}$$

(1c) $$\eta_{T}=\frac{E_{f}/E_{m}-1}{E_{f}/E_{m}+2}$$

where *E_m_*, *E_f_*, and *E_c_* are the moduli of the matrix, the filler, and the composite, respectively, *V_f_* is the fibers’ relative volume concentration, and *l* and *d* are the length and diameter of the fillers. Thus, the composite modulus depends on the filler modulus and on the *l/d* ratio. We assume here: (i) the geometry of both cellulose nanofibers is the one calculated by AFM and (ii) at low nanofiber concentration, the dispersion is homogeneous. From these assumptions, the value of nanofibers modulus as the only variable in Equation (1a) can be calculated by fitting the first four points measured (cellulose and the nanocomposites containing 1%, 5%, and 9% nanocellulose concentration). For lNF nanocomposites, all points yielded the same values of modulus *E_f_* ≈ 80 GPa, which is in agreement with reports of bacterial cellulose and indicates that the assumptions above are reliable [64]. From there, the differences between the model and experimental values, which is seen for higher concentrations, can be explained by fiber agglomerations. It should be pointed out that the modulus reduction, which is not fitted with Equation (1a), even for *l/d* = 1 (spherical-like agglomerates), suggests that for such loading, the homogeneous matrix/filler structure was not maintained and the non-continuous fibers could not bear load properly. Therefore, the model applied here can be considered valid only for low nanocellulose concentrations in which the phenomenon of nanofiber aggregation is not predominant. Similar results of the modeling were obtained for the sNF composites, with a slightly higher value of the fitting modulus (*E_f_* ≈ 90 GPa) and the same discrepancy with experimental data for concentrations above 9%.

Considering other mechanical parameters (Figure 4D–F), yield stress followed a similar trend as Young’s modulus in both families of composites. An initial strong increment (from ~20 MPa for cellulose to ~84 MPa for sNF5 and ~73 MPa for lNF10) was followed by a progressive decline. The trend finished at ~56 MPa for both sNF30 and lNF30 nanocomposites, as agglomeration took place. On the other hand, the elongation at the break showed a twofold increment (from ~3.0% for cellulose to ~7.1 and ~8.5% for sNF and lNF films) that was maintained even at high filler concentrations. This was attributed to the bridging effect of fibers that hinder crack propagation with a toughening effect [65]. Direct measurement of the fracture energy confirmed the improvement from ~37 J/cm^3^ for cellulose to ~449 and ~510 J/cm^3^ for sNF5 and lNF10, respectively, i.e., an increase of ~13 times. These values decreased to ~260 J/cm^3^ for the samples with a 30 wt.% of nanocelluloses.

### 3.4. Water Permeability and Uptake of the Nanocomposites

**Figure 5:**
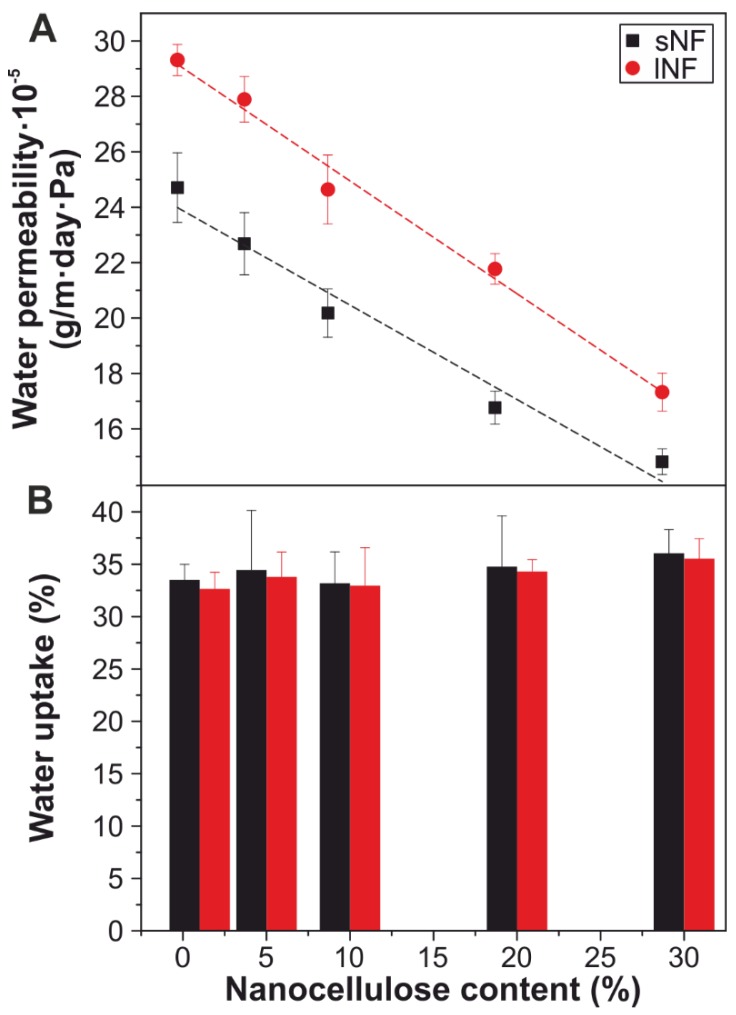
(**A**) Water permeability versus nanocellulose content. (**B**) Water uptake values versus nanocellulose content.

The water permeability was measured for the *all*-cellulose nanocomposites. Figure 5A presents the water permeability values versus the nanocellulose content. Pure cellulose films present a water permeability value of 1.1·10^−3^ g m^−1^ day^−1^ Pa^−1^ (data not shown). When 1 wt.% cellulose nanofibers were added, the values were ~2.5·10^−4^ and 2.9·10^−4^ g m^−1^ day^−1^ Pa^−1^ for sNF1 and lNF1, respectively. Increasing the nanocellulose content, the values decreased linearly until the final values of 1.5·10^−4^ and 1.7·10^−4^ g m^−1^ day^−1^ Pa^−1^ for sNF30 and lNF30, respectively, i.e., a reduction of ~40% for both of them. This decrease can be explained by the increasing tortuosity through the nanocomposite cross-section during water migration. Thus, for the samples containing 1 wt.% nanocellulose, water can easily find a way through the cellulose matrix, which is mainly amorphous [11]. On the other hand, for samples with a 23 wt.% nanocellulose, there are many obstacles—i.e., relatively crystalline, aggregated cellulose nanofibers—that increase the path that water molecules travel to leave the composite. Small differences were found between the two sources of nanocellulose used in this study, being slightly higher values for the films prepared from shorter cellulose nanofibers. This can be explained by a different aggregation and/or folding of these nanocelluloses during the fabrication process, as discussed during the mechanical characterization.

Water uptake was also evaluated for all the samples (Figure 5B). Almost no differences were found with changing the percentage of nanocellulose. The mean water uptake for the nanocomposites was ~34%. This behavior can be explained for the fact that both amorphous cellulose acting as a matrix and nanocelluloses as reinforcements have the typical hydrophilic character of cellulose. Therefore, from a water protection point of view, this material does not provide moisture protection. Nevertheless, further investigations are required to clarify whether water can act as a plasticizer of *all*-cellulose composites in a similar way as described in the literature for other biopolymers [66].

## 4. Conclusions

In this work, we showed that a mixture of TFA and TFAA can be used as a solvent to produce *all*-cellulose nanocomposites from microcrystalline cellulose and cellulose nanofillers (i.e., short and long nanofibers). Cellulose nanofibers were partially dissolved during the production process, increasing the content of the amorphous phase and reducing the crystallite size of cellulose. This allowed good compatibility with the cellulose matrix. The nanocellulose content affected the final properties of the composites: keeping excellent transparency, improving mechanical properties, and relatively reducing the water permeability. These characteristics can be exploited in their potential application as food packaging films.

## Figures and Tables

**Table 1:** Label and final formulation of the samples.

Label	MCC (wt.%)	Nanocellulose	
		Short Nanofibers (wt.%)	Long Nanofibers (wt.%)
**Cellulose**	100	-	-
**sNF1**	99	1	-
**sNF5**	95	5	-
**sNF10**	91	9	-
**sNF20**	83	17	-
**sNF30**	77	23	-
**lNF1**	99	-	1
**lNF5**	95	-	5
**lNF10**	91	-	9
**lNF20**	83	-	17
**lNF30**	77	-	23
